# Supplementary material for: Global transportation infrastructure exposure to the change of precipitation in a warmer world
Source: Nat Commun. 2023 May 3;14:2541. doi: 10.1038/s41467-023-38203-3 (PMC10156714; doi:10.1038/s41467-023-38203-3)
Supplement: Supplementary file 1 — Supplementary Information [file 41467_2023_38203_MOESM1_ESM.pdf]

Supplementary Information for
Global transportation infrastructure exposure to the change of precipitation in a warmer world

Kai Liu[1,2], Qianzhi Wang[1,3], Ming Wang[1], Elco Koks[4]

1 School of National Safety and Emergency Management, Beijing Normal University, China

2 Collaborative Innovation Center on Forecast and Evaluation of Meteorological Disasters (CIC-FEMD), Nanjing University of Information Science & Technology, China

3 School of Systems Science, Beijing Normal University, China

4 Institute for Environmental Studies (IVM), Vrije Universiteit Amsterdam, 1081 HV Amsterdam, Netherlands

The Supplementary information includes:
Supplementary Tables 1-4
Supplementary Figs.1-22

Supplementary Table 1 Design return periods of drainage system of transportation infrastructure

Income groups	Motorway/Trunk/ Primary/Secondary	Tertiary	Railway
Low&Low middle income	2(5)	0(0)	10(20)
Upper middle income	5(10)	2(5)	20(30)
High income	10(20)	5(10)	30(50)

*The assets with design return period of 0 are not be included in further calculation. Values in brackets are higher design standards assumption.

Supplementary Table 2 Climate models used in this study

Models	Country	Institution
ACCESS1-0	Australia	Centre for Australian Weather and Climate Research (CAWCR)
BCC-CSM1-1	China	Beijing Climate Center (BCC)
BNU-ESM	China	Beijing Normal University (BNU)
CCSM4	the United States	National Center for Atmospheric Research (NCAR)
CESM1-BGC	the United States	National Center for Atmospheric Research (NCAR)
CNRM-CM5	France	Centre National de Recherches Meteorologiques- Centre Europeen de Recherche et Formation Avancees en Calcul Scientifique (CNRM-CERFACS)
CSIRO-Mk3-6-0	Australia	Commonwealth Scientific and Industrial Research Organization/Queensland Climate Change Centre of Excellence (CSIRO-QCCCE)
CanESM2	Canada	Canadian Centre for Climate Modelling and Analysis (CCCMA)
GFDL-ESM2G	the United States	Geophysical Fluid Dynamics Laboratory (NOAA-GFDL)
GFDL-ESM2M	the United States	Geophysical Fluid Dynamics Laboratory (NOAA-GFDL)
INM-CM4	Russia	Institute for Numerical Mathematics (INM)
IPSL-CM5A-LR	France	Institut Pierre-Simon Laplace (IPSL)
IPSL-CM5A-MR	France	Institut Pierre-Simon Laplace (IPSL)
MIROC-ESM-CHEM	Japan	Atmosphere and Ocean Research Institute (The University of Tokyo), National Institute for Environmental Studies, and Japan Agency for Marine-Earth Science and Technology (MIROC)
MIROC-ESM	Japan	Atmosphere and Ocean Research Institute (The University of Tokyo), National Institute for Environmental Studies, and Japan Agency for Marine-Earth Science and Technology (MIROC)
MIROC5	Japan	Atmosphere and Ocean Research Institute (The University of Tokyo), National Institute for Environmental Studies, and Japan Agency for Marine-Earth Science and Technology (MIROC)
MPI-ESM-LR	Germany	Max Planck Institute for Meteorology (MPI-M)
MPI-ESM-MR	Germany	Max Planck Institute for Meteorology (MPI-M)
MRI-CGCM3	Japan	Meteorological Research Institute (MRI)
NorESM1-M	Norway	Norwegian Climate Centre (NCC)

Supplementary Table 3 Transportation assets used in this study

Category	Class	Description	Length (km)
Road	Motorway	A restricted access major divided highway, normally with 2 or more running lanes plus emergency hard shoulder. Equivalent to the Freeway, Autobahn, etc..	1,139,209
	Trunk	The most important roads in a country's system that aren't motorways. (Need not necessarily be a divided highway.)	1,959,390
	Primary	The next most important roads in a country's system. (Often link larger towns.)	3,035,790
	Secondary	The next most important roads in a country's system. (Often link towns.)	4,638,696
	Tertiary	The next most important roads in a country's system. (Often link smaller towns and villages)	9,050,024
Railway	Rail	Full sized passenger or freight trains in the standard gauge for the country or state.	3,788,416

Supplementary Table 4 Relations between return period change and exceedance probability change

Decease of Return Period	Increase of Exceedance Probability
0%	11%
15%	18%
20%	25%
25%	33%
30%	43%
35%	53%
40%	67%
45%	82%
50%	100%

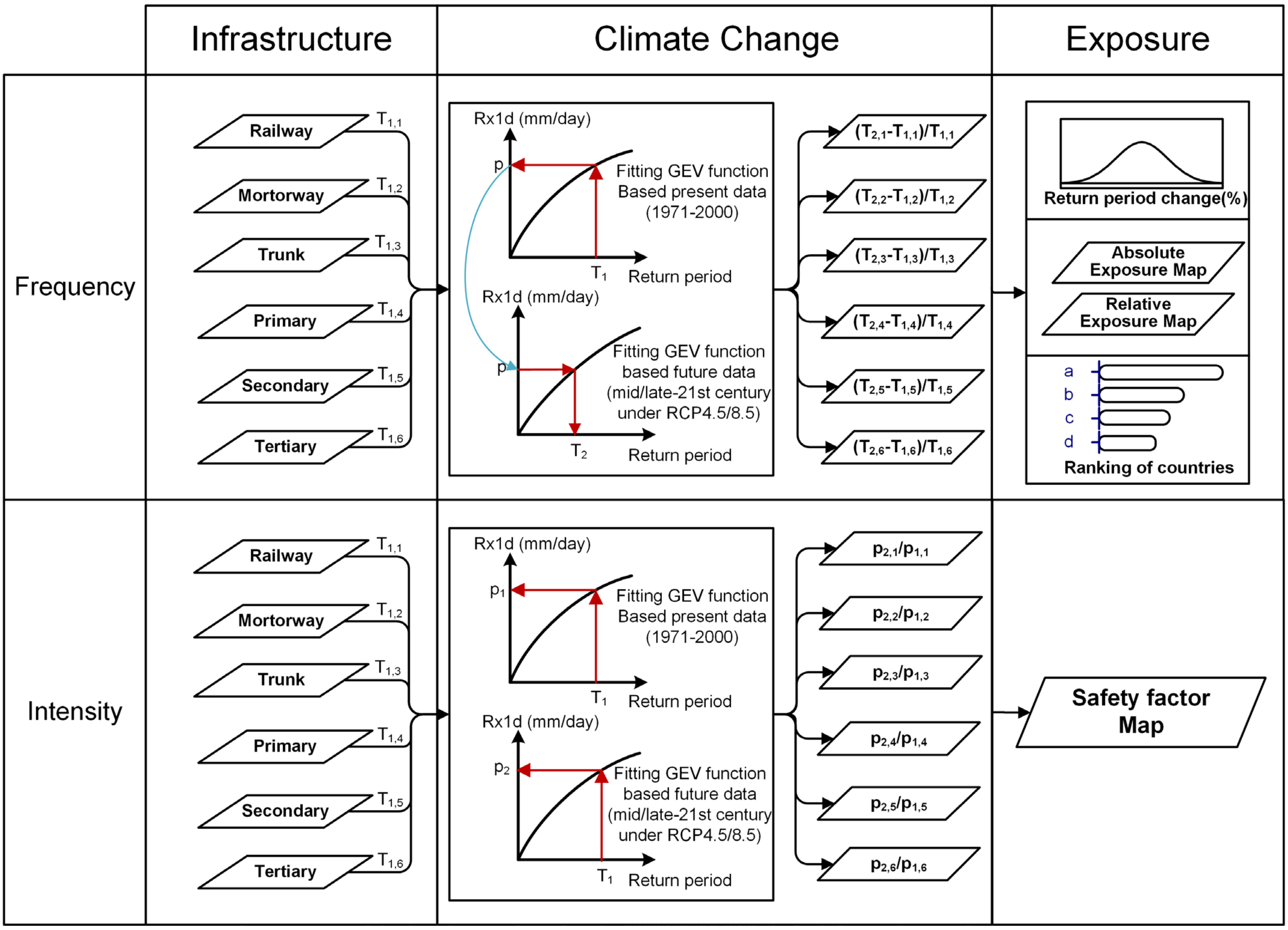

Supplementary Fig.1. Methodology

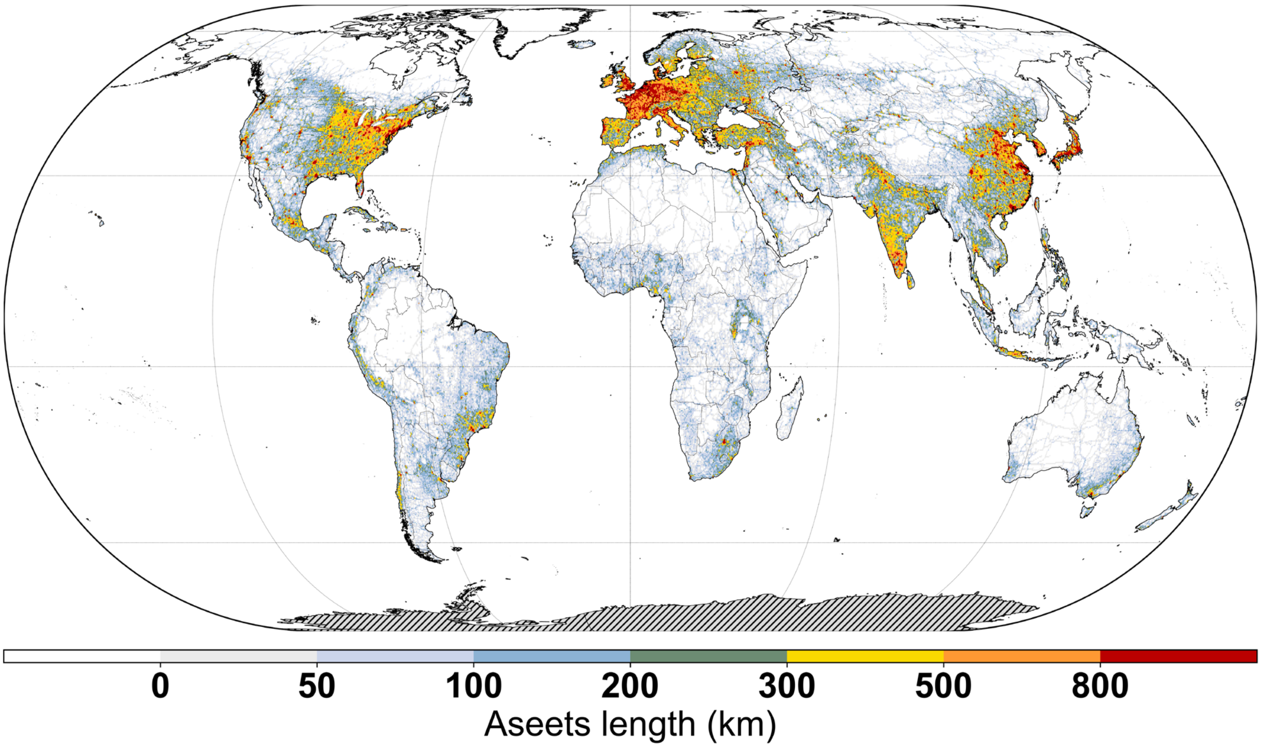

Supplementary Fig.2. Spatial distribution of global railway and road assets.

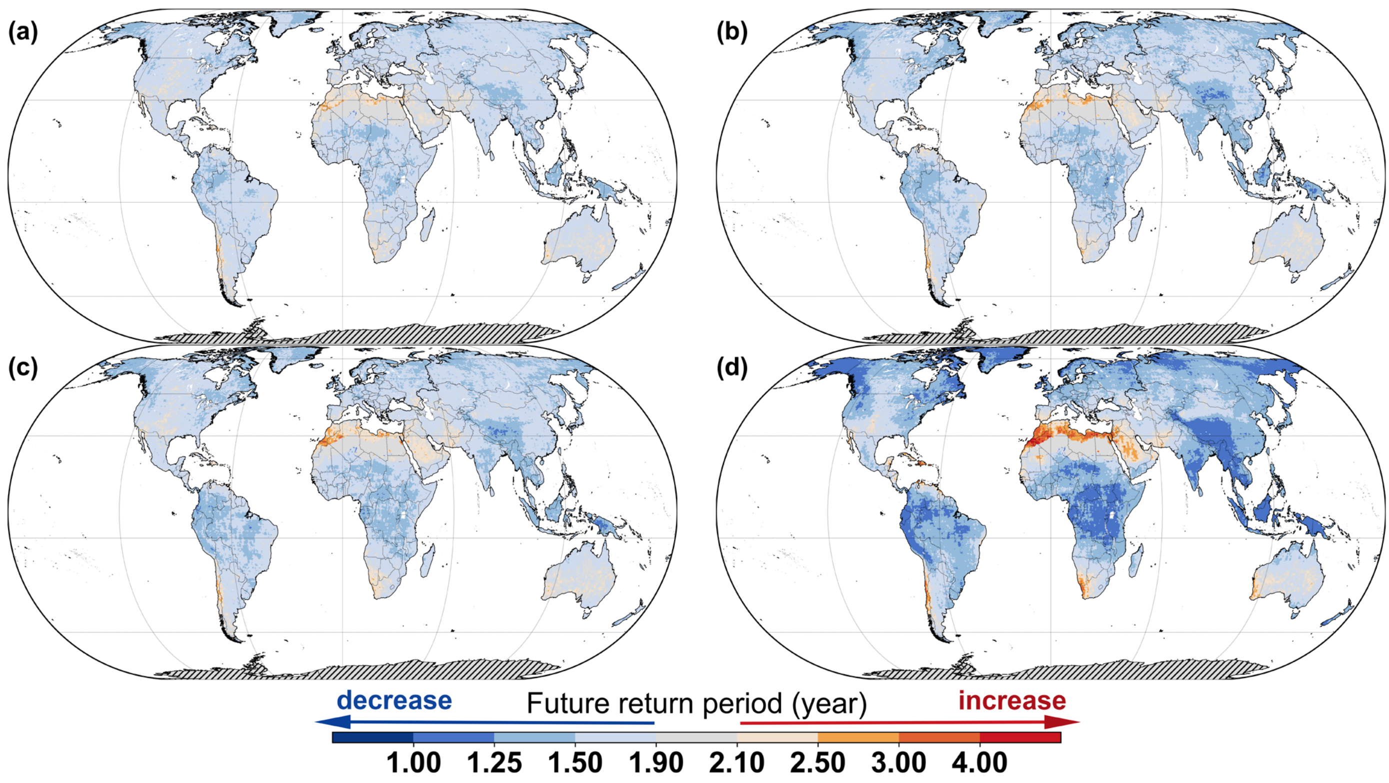

Supplementary Fig.3. Multi-model median return period for precipitation in the future for 1-in-2-year precipitation compared with the period of 1971–2000: (a) mid-21st century (2030-2059) under the RCP4.5 scenario; (b) late-21st century (2070-2099) under the RCP4.5 scenario; (c) mid-21st century (2030-2059) under the RCP8.5 scenario; and (d) late-21st century (2070-2099) under the RCP8.5 scenario.

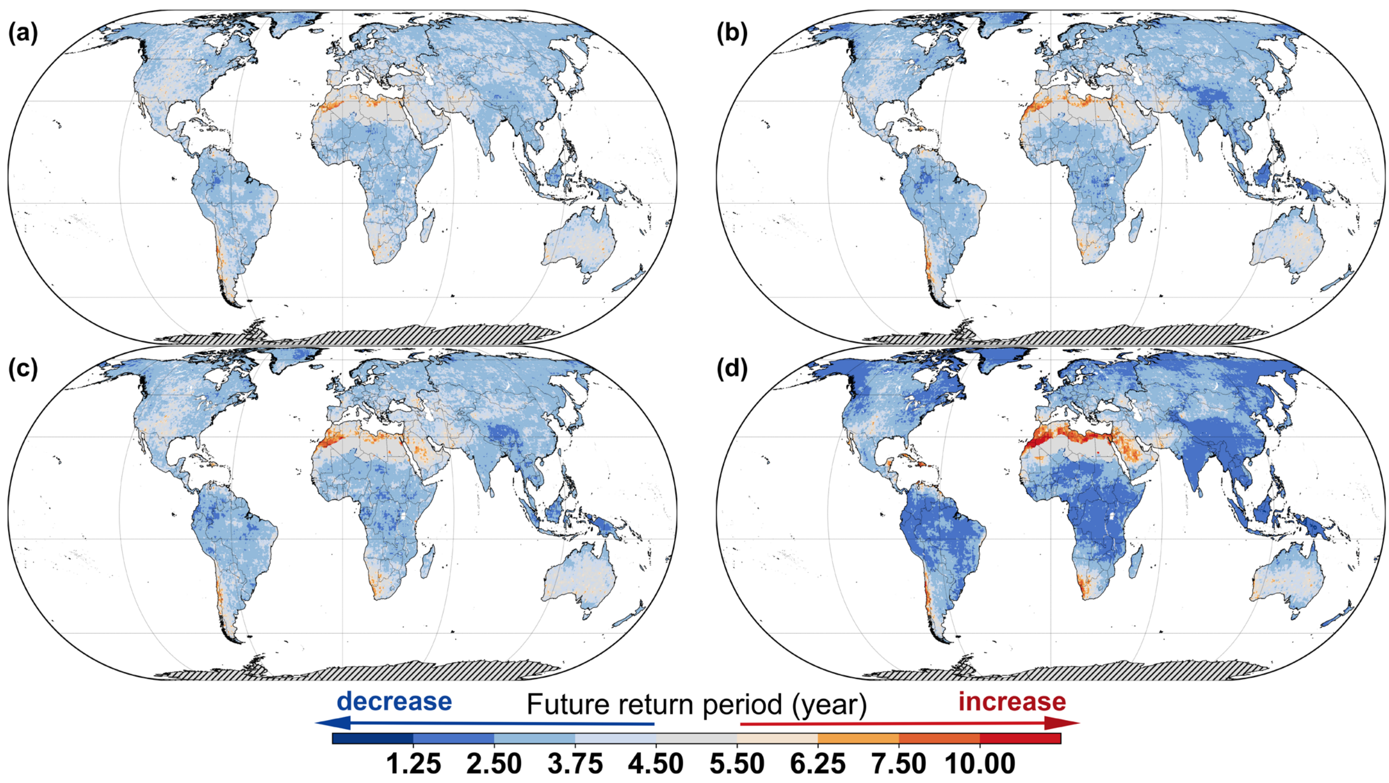

Supplementary Fig.4. Multi-model median return period for precipitation in the future for 1-in-5-year precipitation compared with the period of 1971–2000: (a) mid-21st century (2030-2059) under the RCP4.5 scenario; (b) late-21st century (2070-2099) under the RCP4.5 scenario; (c) mid-21st century (2030-2059) under the RCP8.5 scenario; and (d) late-21st century (2070-2099) under the RCP8.5 scenario.

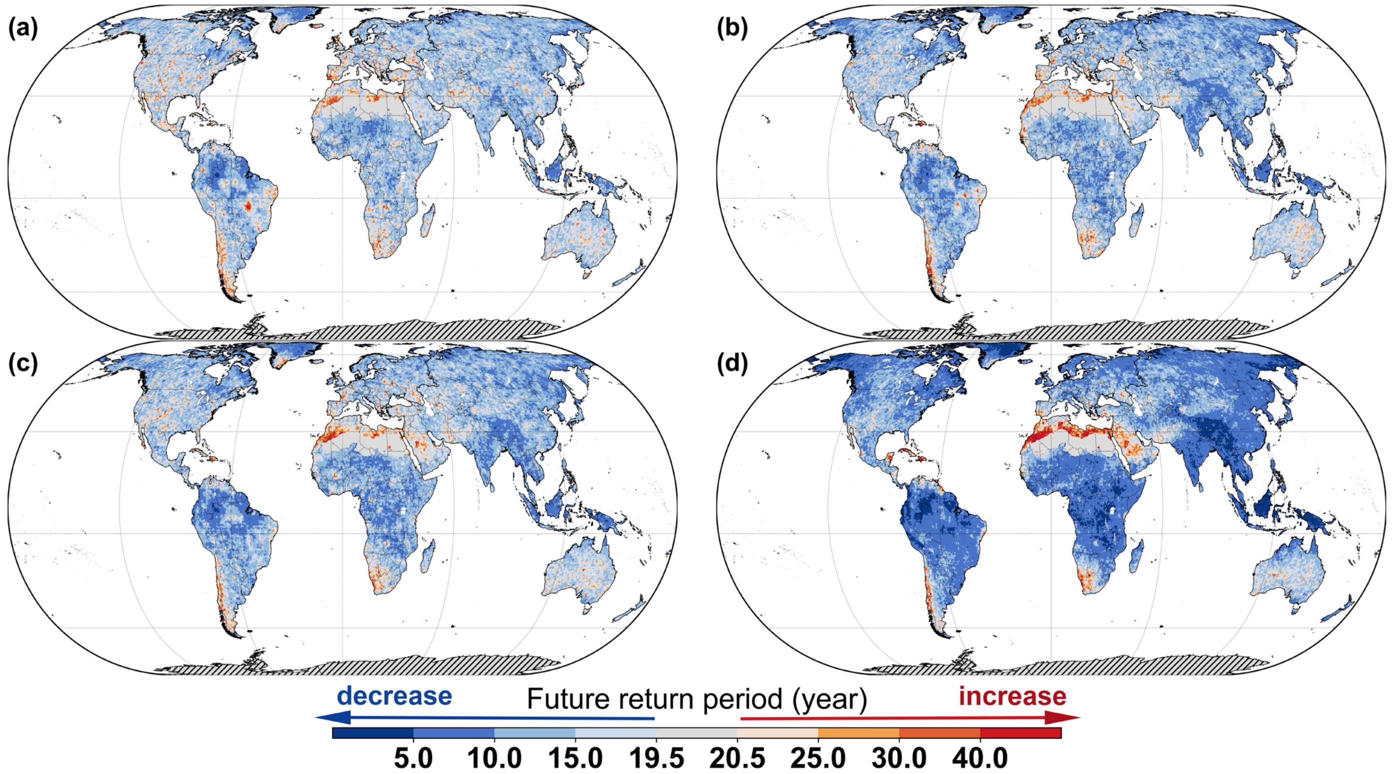

Supplementary Fig.5. Multi-model median return period for precipitation in the future for 1-in-20-year precipitation compared with the period of 1971–2000: (a) mid-21st century (2030-2059) under the RCP4.5 scenario; (b) late-21st century (2070-2099) under the RCP4.5 scenario; (c) mid-21st century (2030-2059) under the RCP8.5 scenario; and (d) late-21st century (2070-2099) under the RCP8.5 scenario.

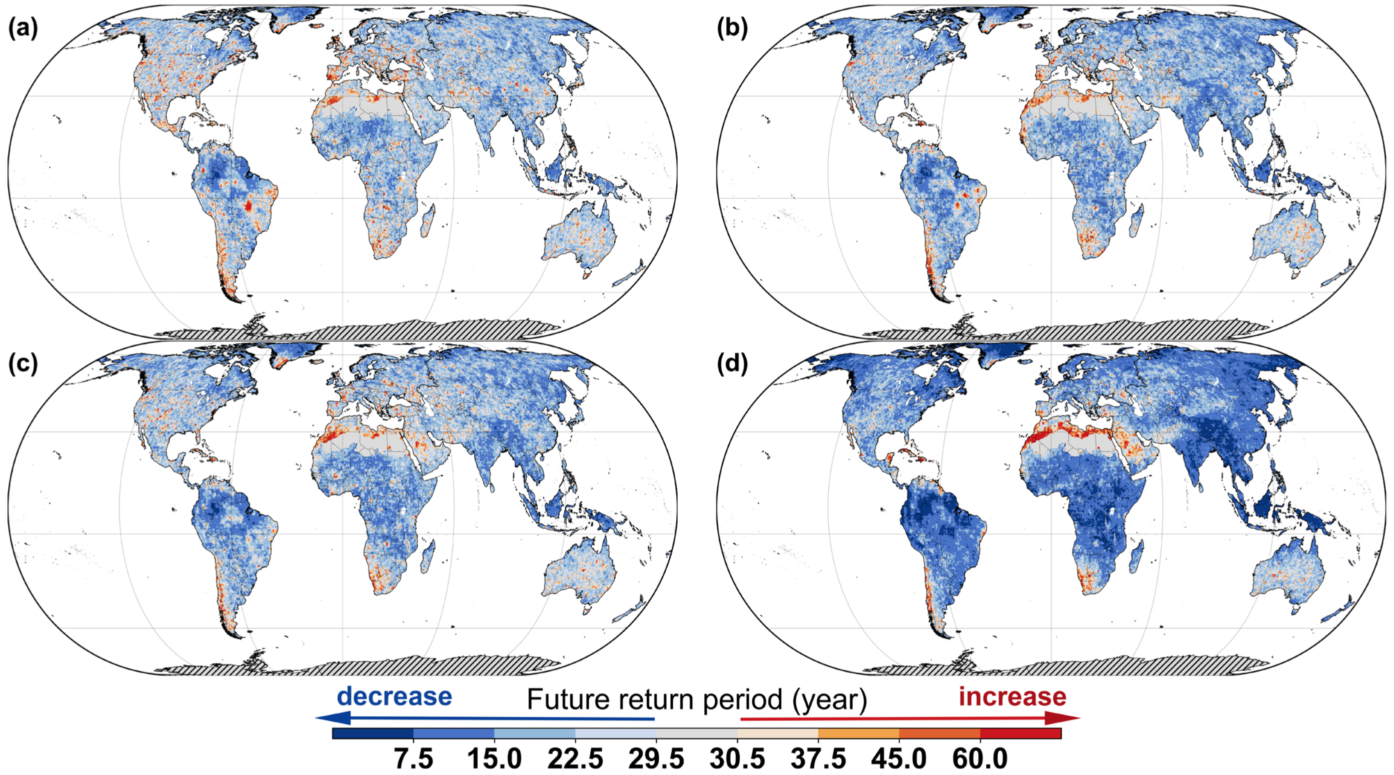

Supplementary Fig.6. Multi-model median return period for precipitation in the future for 1-in-30 year precipitation compared with the period of 1971–2000: (a) mid-21st century (2030-2059) under the RCP4.5 scenario; (b) late-21st century (2070-2099) under the RCP4.5 scenario; (c) mid-21st century (2030-2059) under the RCP8.5 scenario; and (d) late-21st century (2070-2099) under the RCP8.5 scenario.

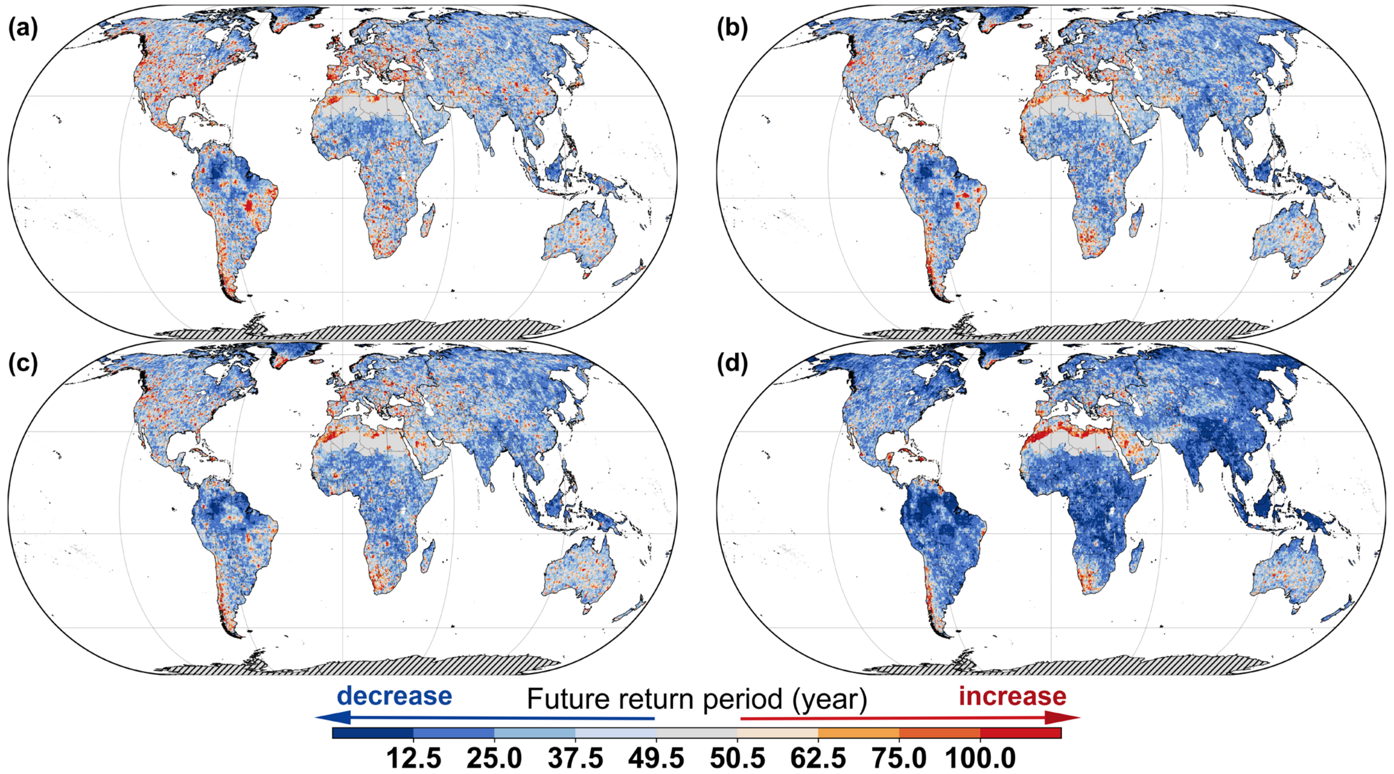

Supplementary Fig.7. Multi-model median return period for precipitation in the future for 1-in-50-year precipitation compared with the period of 1971–2000: (a) mid-21st century (2030-2059) under the RCP4.5 scenario; (b) late-21st century (2070-2099) under the RCP4.5 scenario; (c) mid-21st century (2030-2059) under the RCP8.5 scenario; and (d) late-21st century (2070-2099) under the RCP8.5 scenario.

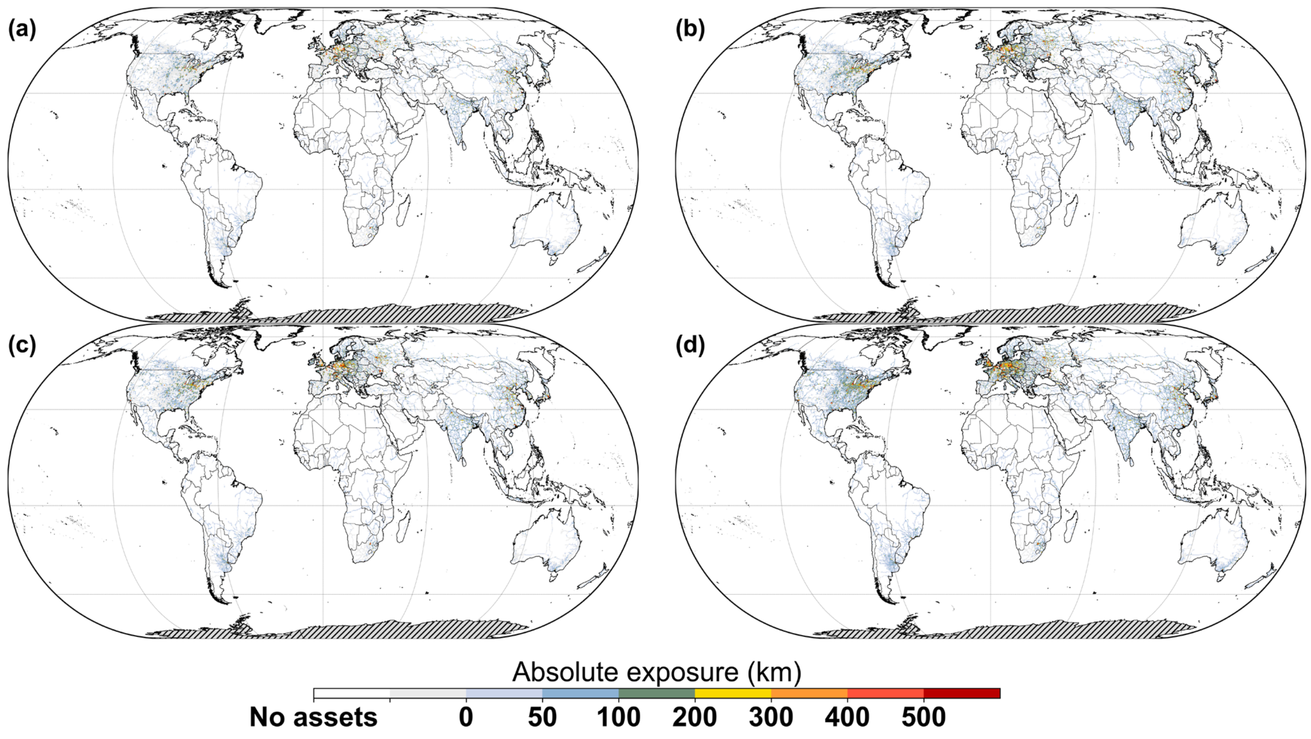

Supplementary Fig.8. Spatial distribution of absolute exposure of global railway assets under different time periods and scenarios: (a) mid-21st century (2030-2059) under the RCP4.5 scenario; (b) late-21st century (2070-2099) under the RCP4.5 scenario; (c) mid-21st century (2030-2059) under the RCP8.5 scenario; and (d) late-21st century (2070-2099) under the RCP8.5 scenario. Results are shown in a grid size of approximately 25 km × 25 km. The absolute exposure is defined as the total length of railway assets within a grid exposed to a more than 25% decrease in the design return period in future.

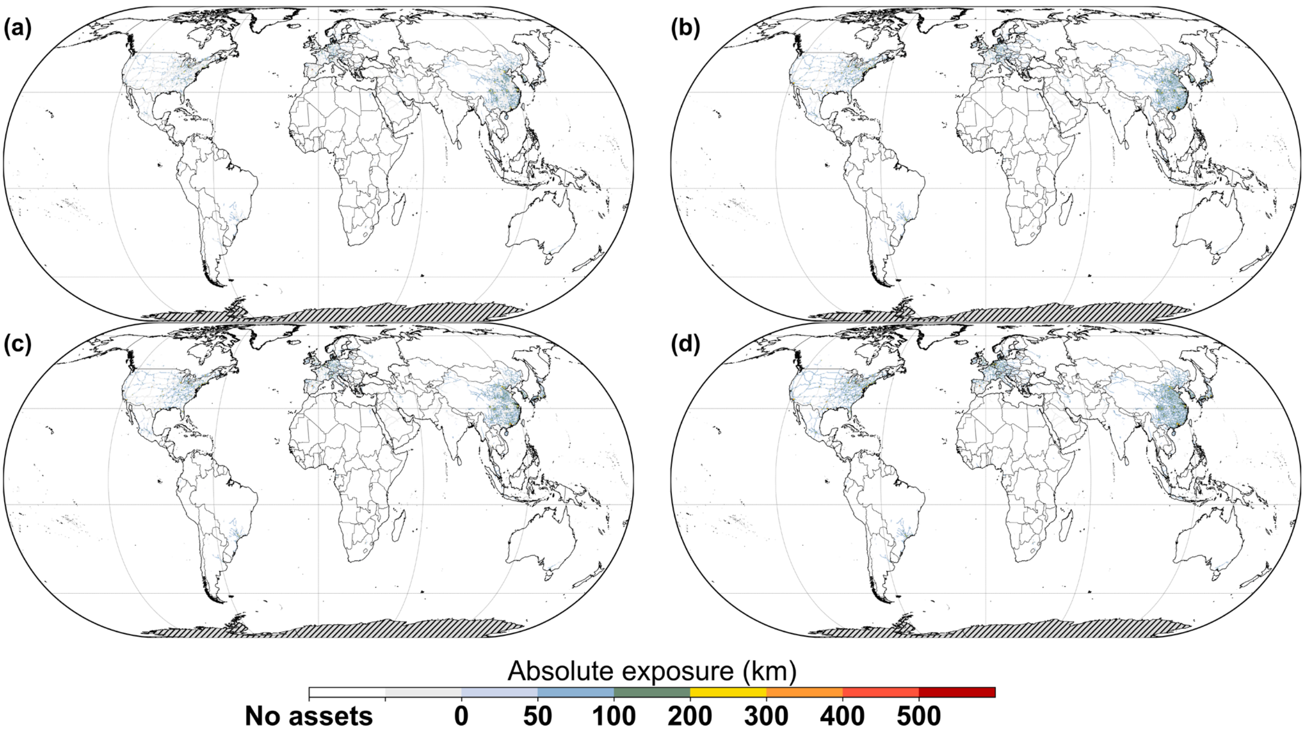

Supplementary Fig.9. Spatial distribution of absolute exposure of global motorway assets under different time periods and scenarios: (a) mid-21st century (2030-2059) under the RCP4.5 scenario; (b) late-21st century (2070-2099) under the RCP4.5 scenario; (c) mid-21st century (2030-2059) under the RCP8.5 scenario; and (d) late-21st century (2070-2099) under the RCP8.5 scenario. Results are shown in a grid size of approximately 25 km × 25 km. The absolute exposure is defined as the total length of motorway assets within a grid exposed to a more than 25% decrease in the design return period in future.

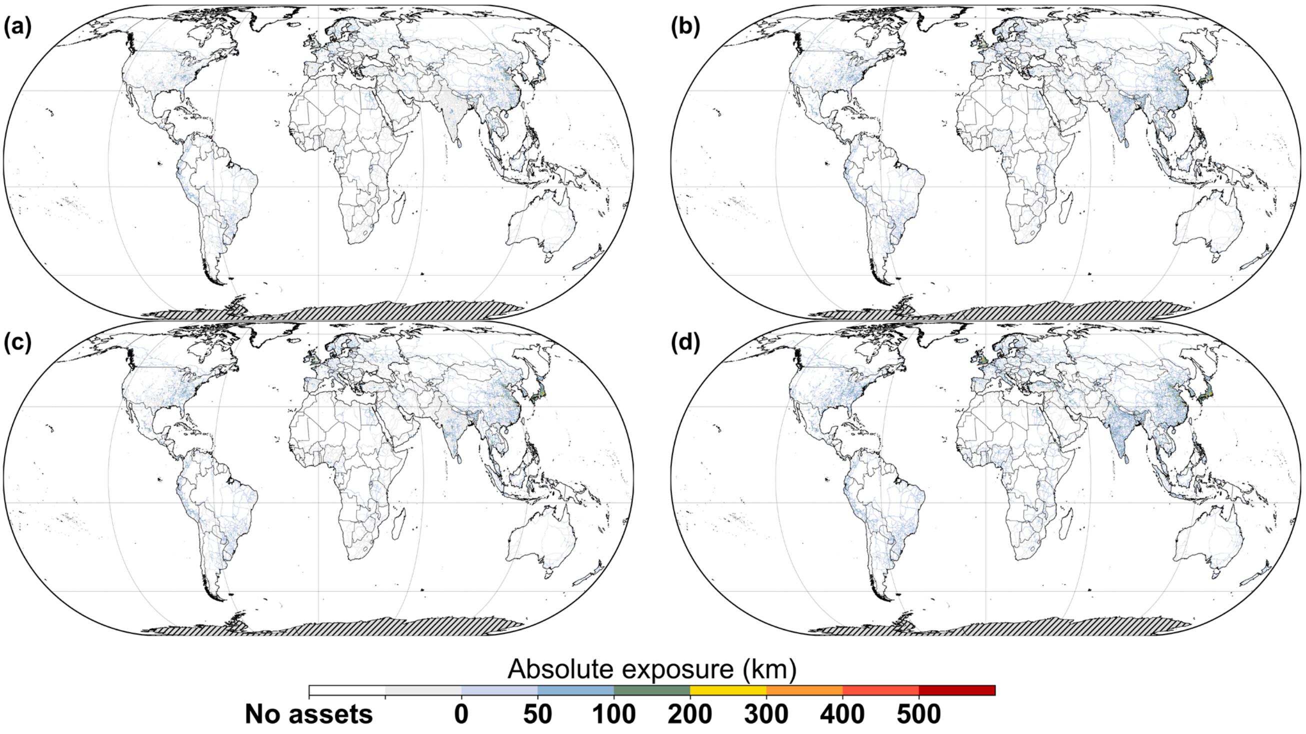

Supplementary Fig.10. Spatial distribution of absolute exposure of global trunk assets under different time periods and scenarios: (a) mid-21st century (2030-2059) under the RCP4.5 scenario; (b) late-21st century (2070-2099) under the RCP4.5 scenario; (c) mid-21st century (2030-2059) under the RCP8.5 scenario; and (d) late-21st century (2070-2099) under the RCP8.5 scenario. Results are shown in a grid size of approximately 25 km × 25 km. The absolute exposure is defined as the total length of trunk assets within a grid exposed to a more than 25% decrease in the design return period in future.

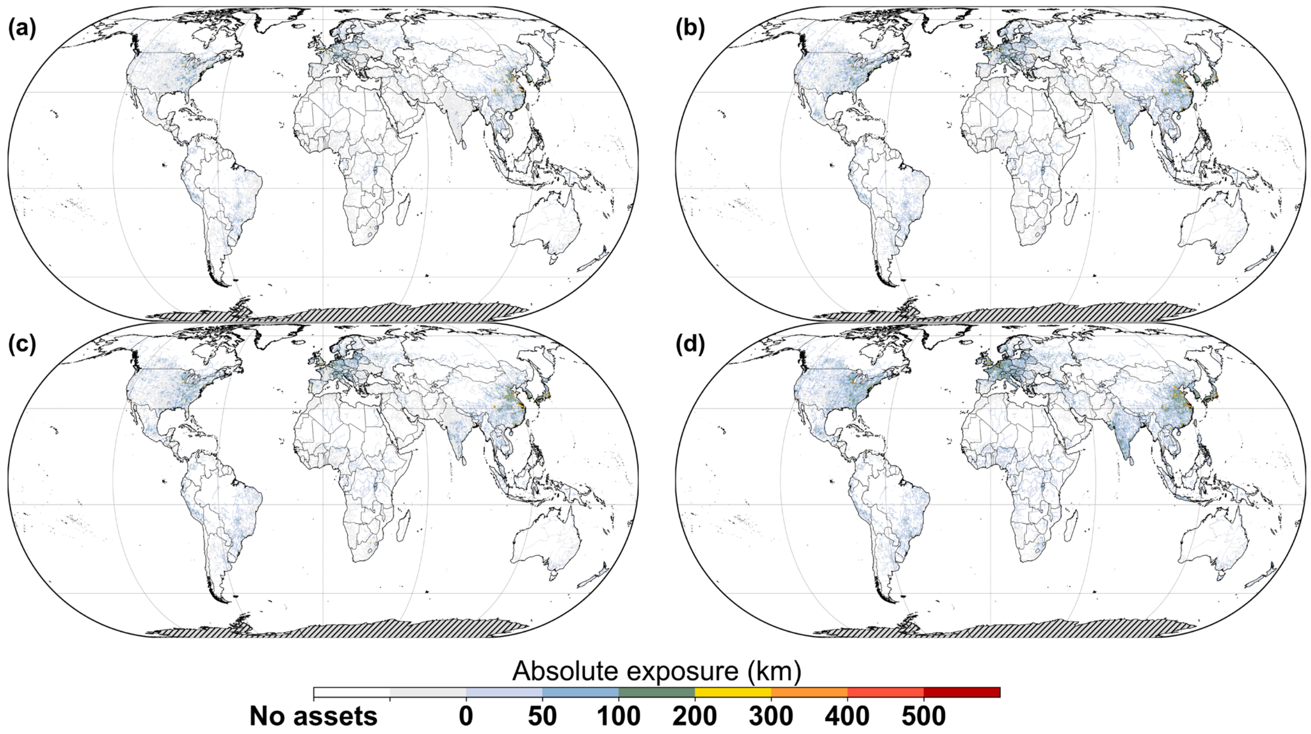

Supplementary Fig.11. Spatial distribution of absolute exposure of global primary road assets under different time periods and scenarios: (a) mid-21st century (2030-2059) under the RCP4.5 scenario; (b) late-21st century (2070-2099) under the RCP4.5 scenario; (c) mid-21st century (2030-2059) under the RCP8.5 scenario; and (d) late-21st century (2070-2099) under the RCP8.5 scenario. Results are shown in a grid size of approximately 25 km × 25 km. The absolute exposure is defined as the total length of primary road assets within a grid exposed to a more than 25% decrease in the design return period in future.

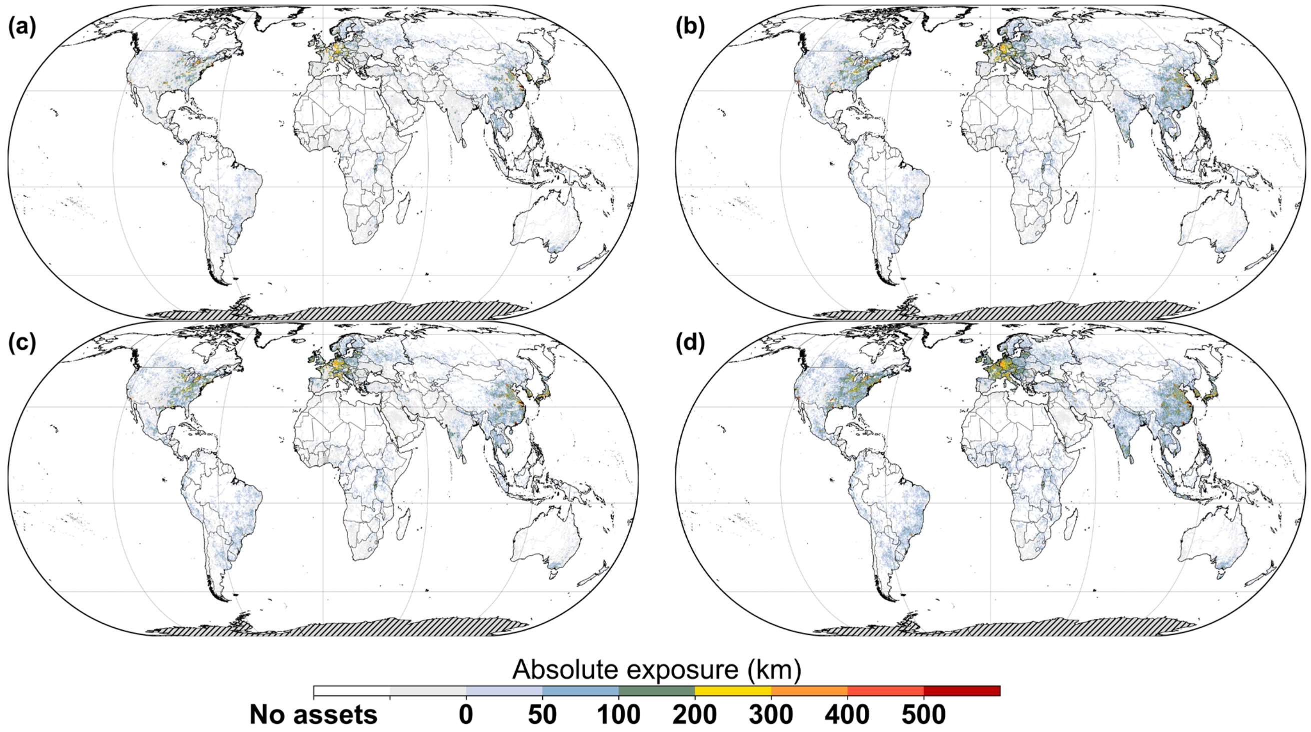

Supplementary Fig.12. Spatial distribution of absolute exposure of global secondary road assets under different time periods and scenarios: (a) mid-21st century (2030-2059) under the RCP4.5 scenario; (b) late-21st century (2070-2099) under the RCP4.5 scenario; (c) mid-21st century (2030-2059) under the RCP8.5 scenario; and (d) late-21st century (2070-2099) under the RCP8.5 scenario. Results are shown in a grid size of approximately 25 km × 25 km. The absolute exposure is defined as the total length of secondary road assets within a grid exposed to a more than 25% decrease in the design return period in future.

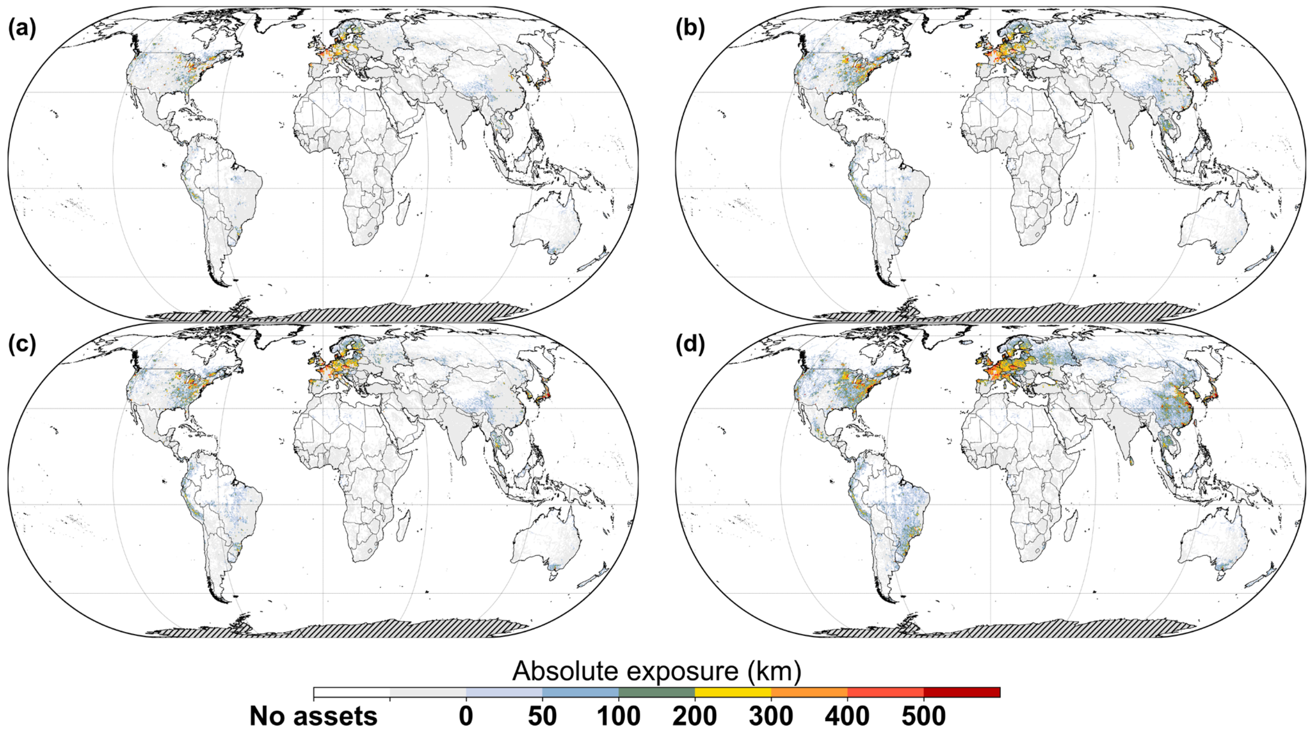

Supplementary Fig.13. Spatial distribution of absolute exposure of global tertiary road assets under different time periods and scenarios: (a) mid-21st century (2030-2059) under the RCP4.5 scenario; (b) late-21st century (2070-2099) under the RCP4.5 scenario; (c) mid-21st century (2030-2059) under the RCP8.5 scenario; and (d) late-21st century (2070-2099) under the RCP8.5 scenario. Results are shown in a grid size of approximately 25 km × 25 km. The absolute exposure is defined as the total length of tertiary road assets within a grid exposed to a more than 25% decrease in the design return period in future.

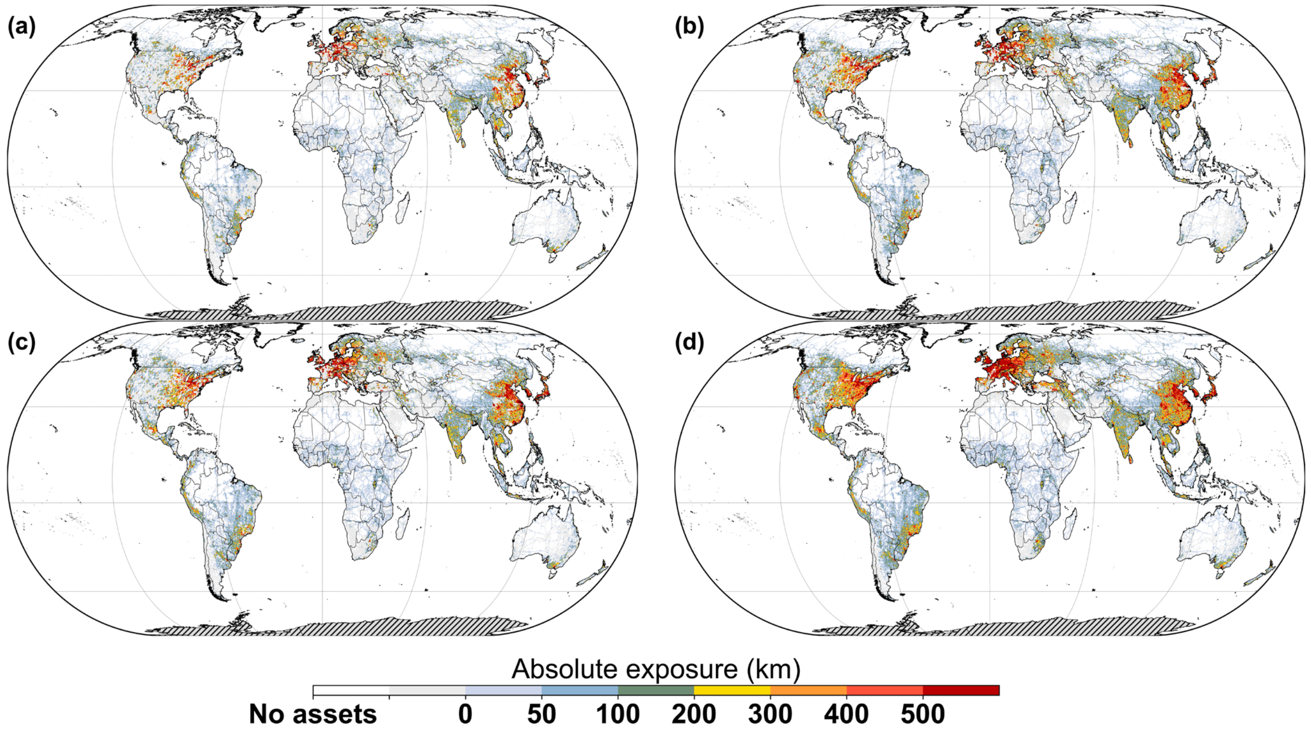

Supplementary Fig.14. Spatial distribution of absolute exposure of global road and railway assets under different time periods and scenarios (based higher current design standards assumption): (a) mid-21st century(2030-2059) under the RCP4.5 scenario; (b) late-21st century(2070-2099) under the RCP4.5 scenario; (c) mid-21st century(2030-2059) under the RCP8.5 scenario; and (d) late-21st century(2070-2099) under the RCP8.5 scenario. Results are shown in a grid size of approximately 25 km × 25 km. The absolute exposure is defined as the total length of road and railway assets within a grid exposed to a more than 25% decrease in the design return period in future.

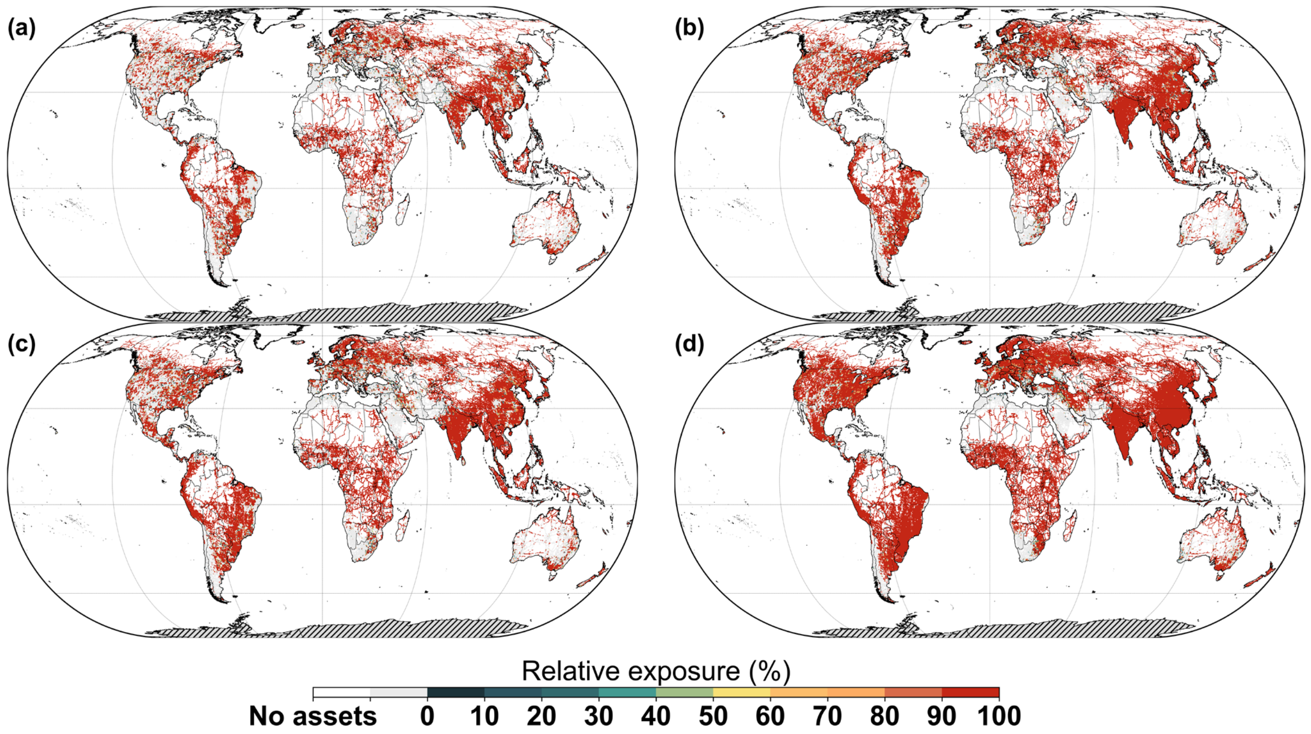

Supplementary Fig.15. Spatial distribution of relative exposure of global road and railway assets under different time periods and scenarios (based higher current design standards assumption): (a) mid-21st century(2030-2059) under the RCP4.5 scenario; (b) late-21st century(2070-2099) under the RCP4.5 scenario; (c) mid-21st century(2030-2059) under the RCP8.5 scenario; and (d) late-21st century(2070-2099) under the RCP8.5 scenario. Results are shown in a grid size of approximately 25 km × 25 km. The relative exposure is defined as the ratio of the absolute exposure to the total assets within a gird.

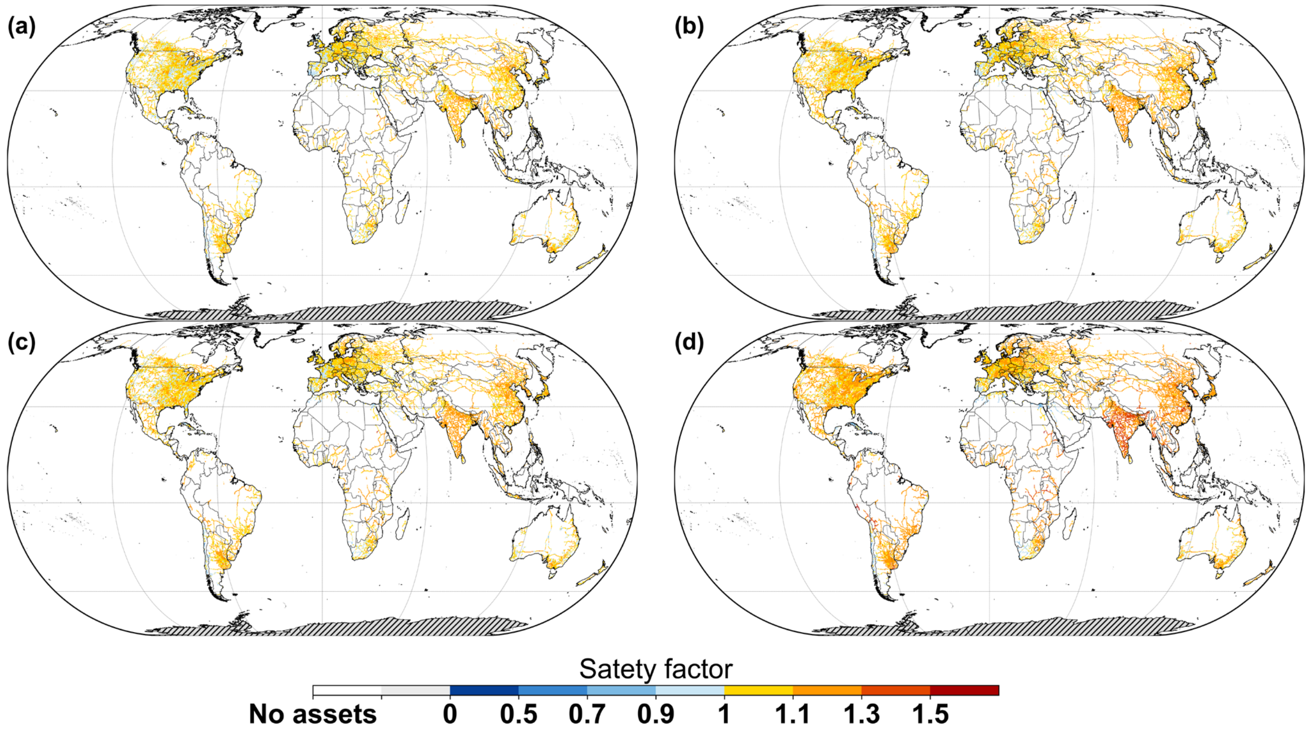

Supplementary Fig.16. Safety factor for climate change adaptation of global railway assets with respect to current design under different time periods and scenarios: (a) mid-21st century (2030-2059) under the RCP4.5 scenario; (b) late-21st century (2070-2099) under the RCP4.5 scenario; (c) mid-21st century (2030-2059) under the RCP8.5 scenario; and (d) late-21st century (2070-2099) under the RCP8.5 scenario. Results are shown in a grid size of approximately 25 km × 25 km. A value of 1 indicates no changes in the designed precipitation, a value of 1.2, for example, means a factor of 1.2 is suggested to apply to the designed precipitation intensity.

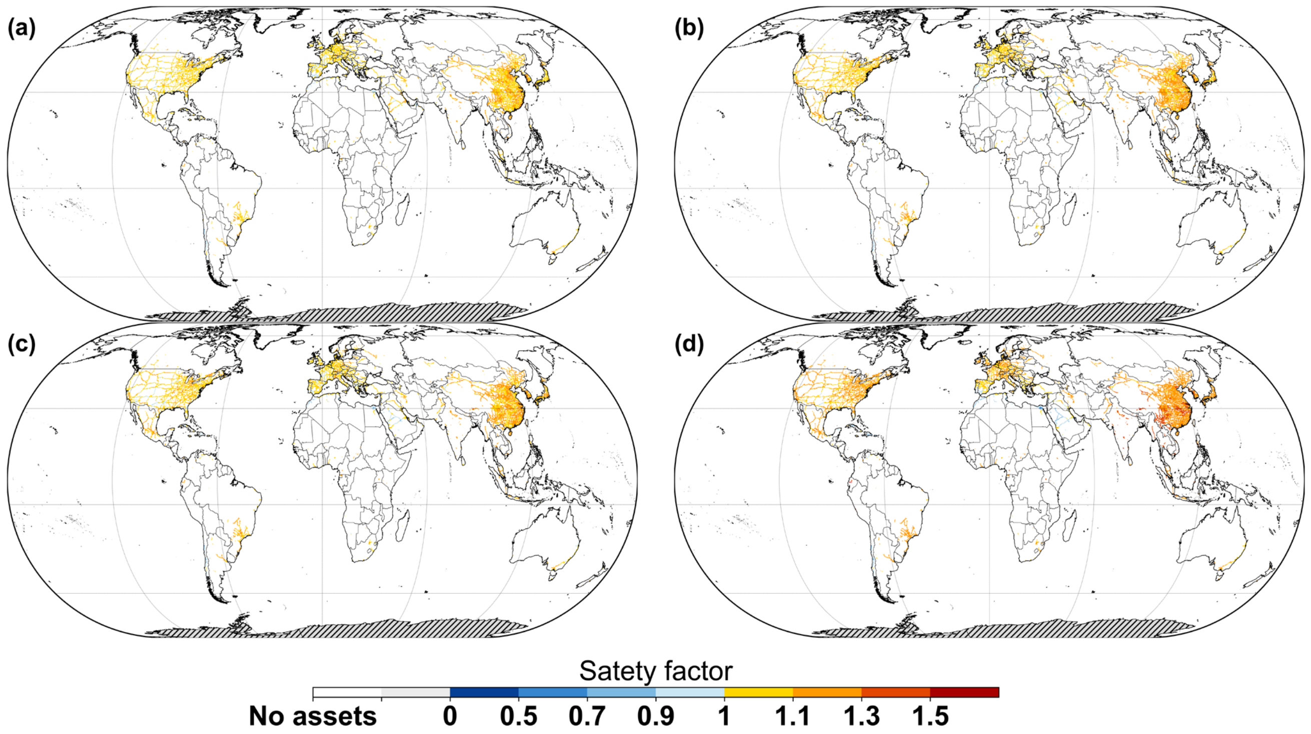

Supplementary Fig.17. Safety factor for climate change adaptation of global motorway assets with respect to current design under different time periods and scenarios: (a) mid-21st century (2030-2059) under the RCP4.5 scenario; (b) late-21st century (2070-2099) under the RCP4.5 scenario; (c) mid-21st century (2030-2059) under the RCP8.5 scenario; and (d) late-21st century (2070-2099) under the RCP8.5 scenario. Results are shown in a grid size of approximately 25 km × 25 km. A value of 1 indicates no changes in the designed precipitation, a value of 1.2, for example, means a factor of 1.2 is suggested to apply to the designed precipitation intensity.

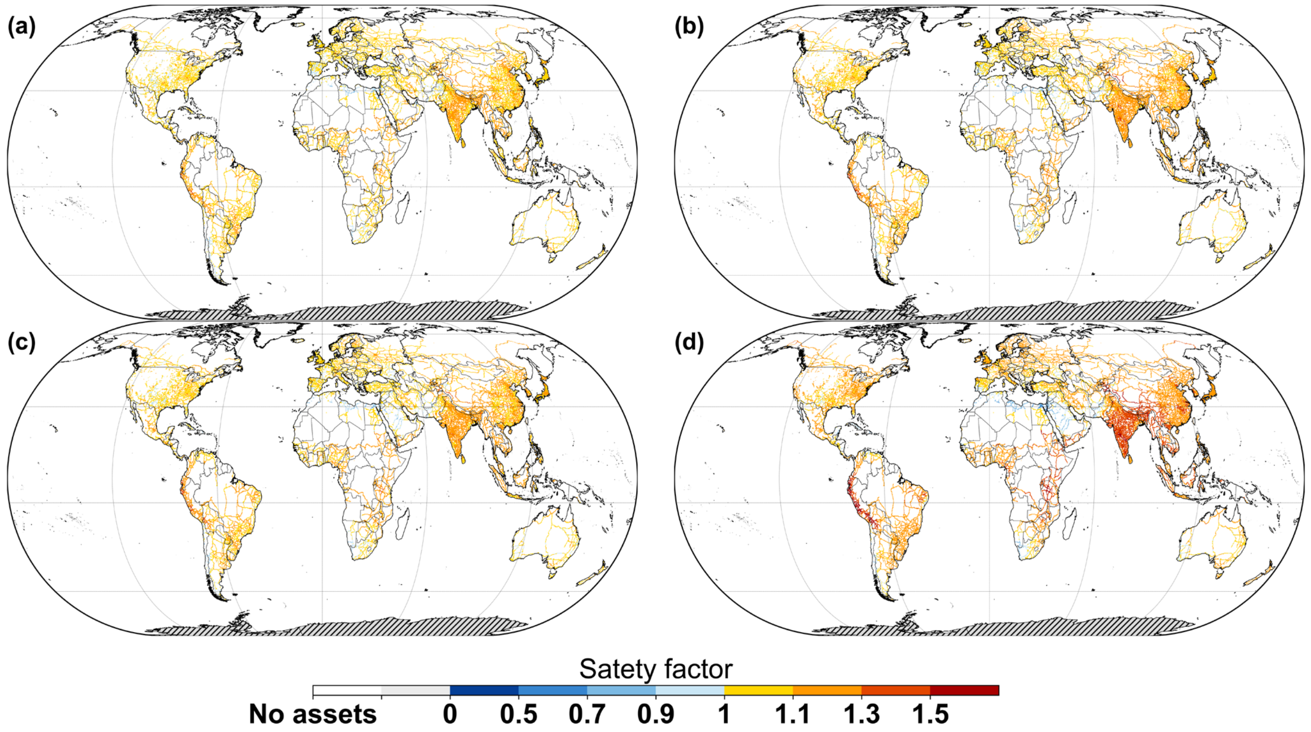

Supplementary Fig.18. Safety factor for climate change adaptation of global trunk assets with respect to current design under different time periods and scenarios: (a) mid-21st century (2030-2059) under the RCP4.5 scenario; (b) late-21st century (2070-2099) under the RCP4.5 scenario; (c) mid-21st century (2030-2059) under the RCP8.5 scenario; and (d) late-21st century (2070-2099) under the RCP8.5 scenario. Results are shown in a grid size of approximately 25 km × 25 km. A value of 1 indicates no changes in the designed precipitation, a value of 1.2, for example, means a factor of 1.2 is suggested to apply to the designed precipitation intensity.

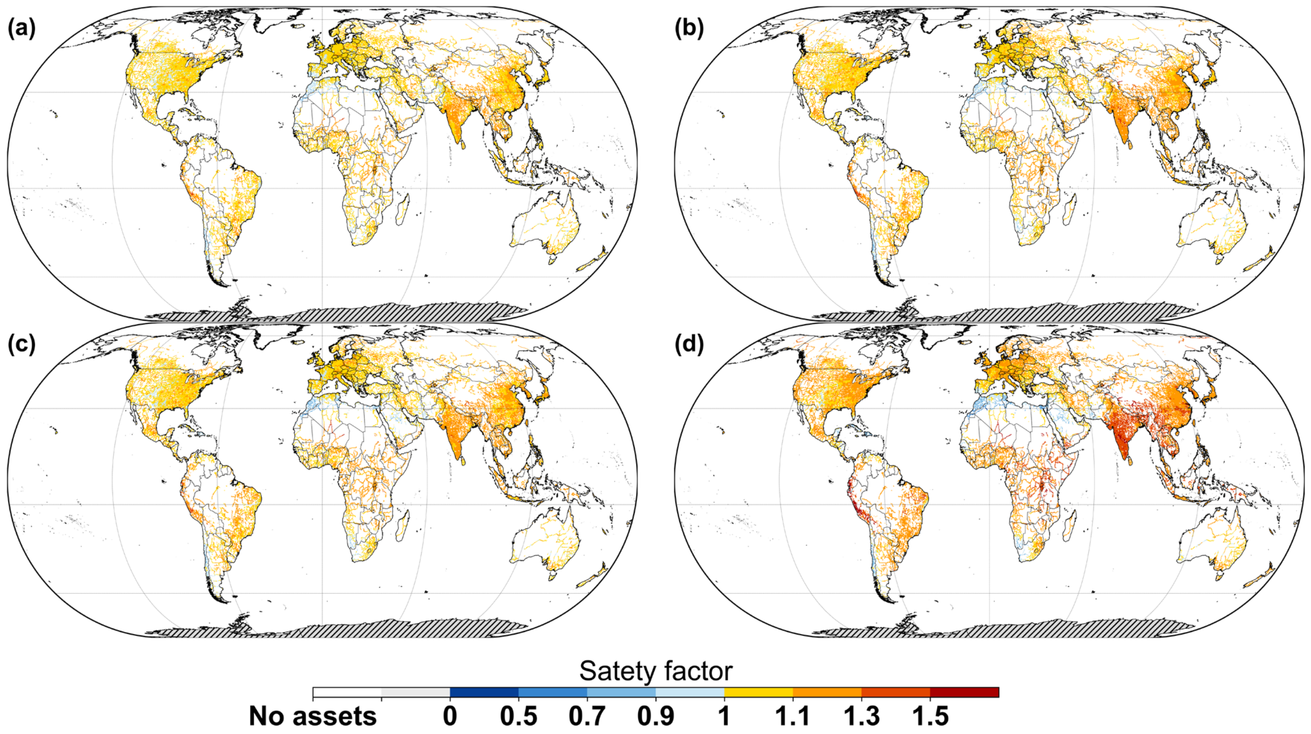

Supplementary Fig.19. Safety factor for climate change adaptation of global primary road assets with respect to current design under different time periods and scenarios: (a) mid-21st century (2030-2059) under the RCP4.5 scenario; (b) late-21st century (2070-2099) under the RCP4.5 scenario; (c) mid-21st century (2030-2059) under the RCP8.5 scenario; and (d) late-21st century (2070-2099) under the RCP8.5 scenario. Results are shown in a grid size of approximately 25 km × 25 km. A value of 1 indicates no changes in the designed precipitation, a value of 1.2, for example, means a factor of 1.2 is suggested to apply to the designed precipitation intensity.

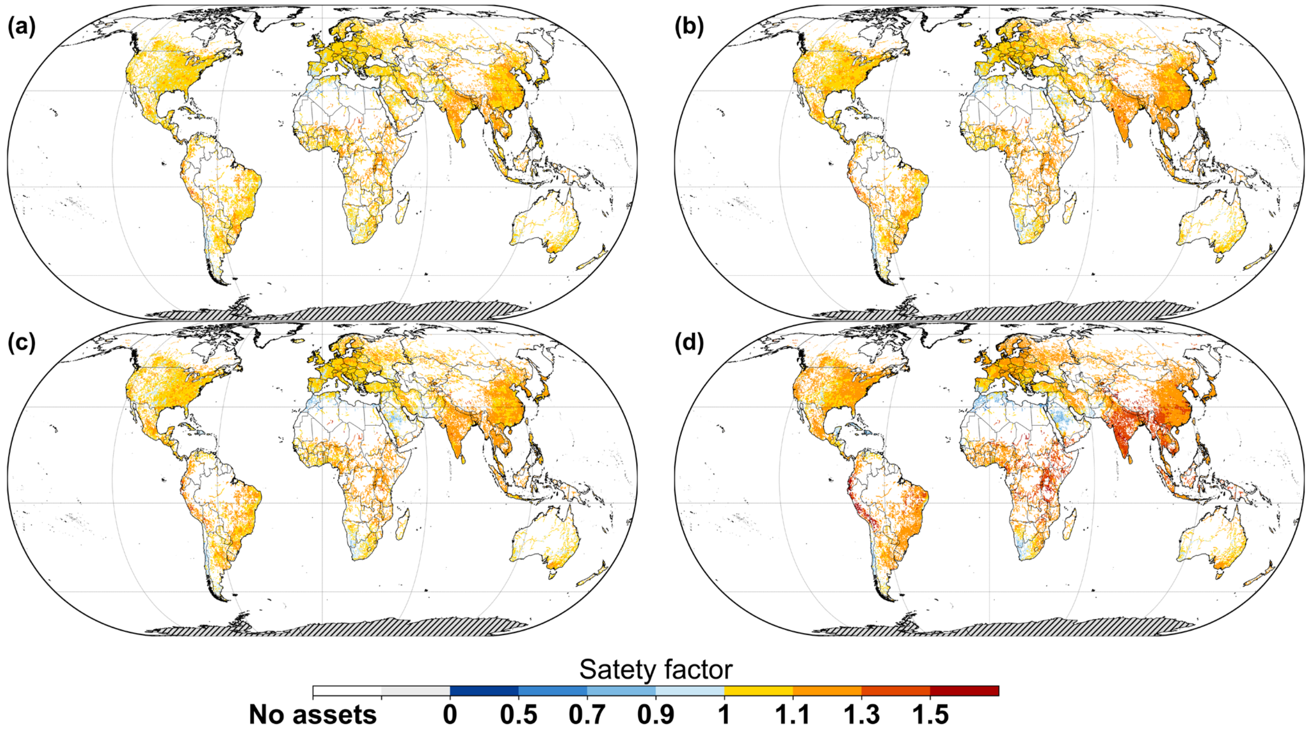

Supplementary Fig.20. Safety factor for climate change adaptation of global secondary roads with respect to current design under different time periods and scenarios: (a) mid-21st century (2030-2059) under the RCP4.5 scenario; (b) late-21st century (2070-2099) under the RCP4.5 scenario; (c) mid-21st century (2030-2059) under the RCP8.5 scenario; and (d) late-21st century (2070-2099) under the RCP8.5 scenario. Results are shown in a grid size of approximately 25 km × 25 km. A value of 1 indicates no changes in the designed precipitation, a value of 1.2, for example, means a factor of 1.2 is suggested to apply to the designed precipitation intensity.

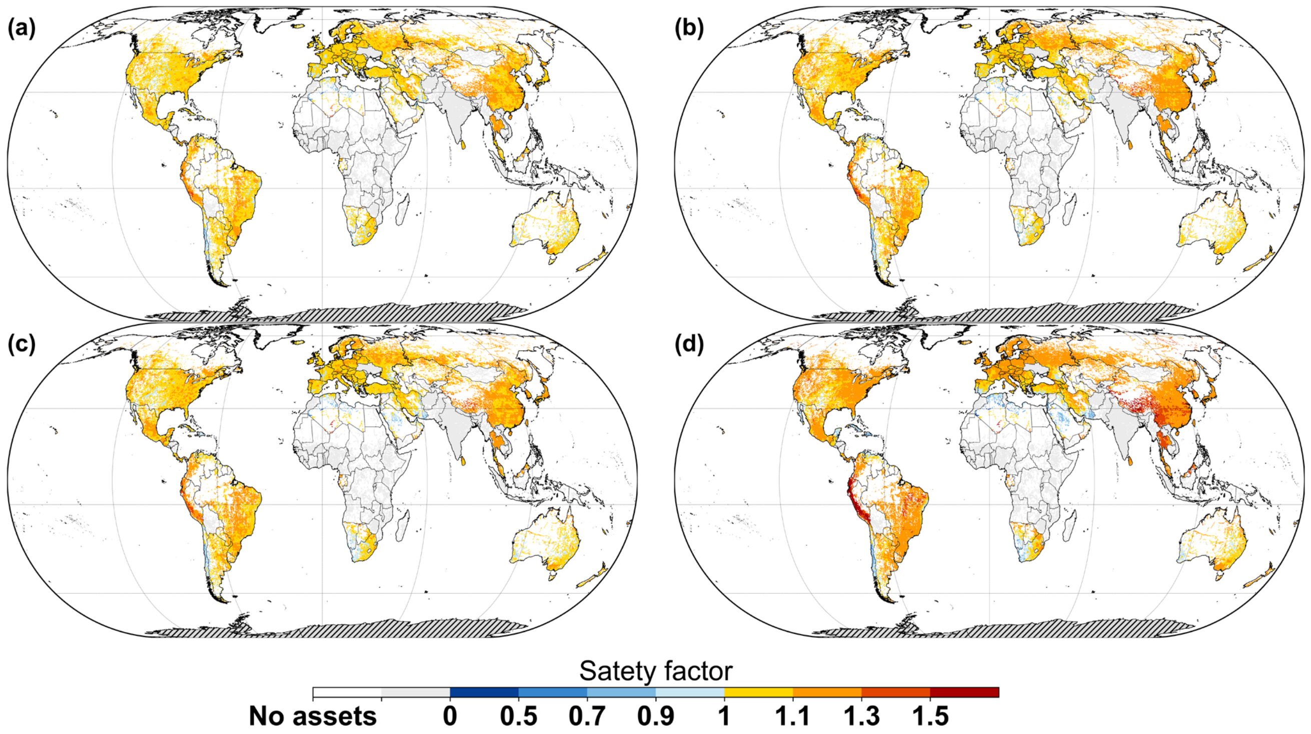

Supplementary Fig.21. Safety factor for climate change adaptation of global tertiary road assets with respect to current design under different time periods and scenarios: (a) mid-21st century (2030-2059) under the RCP4.5 scenario; (b) late-21st century (2070-2099) under the RCP4.5 scenario; (c) mid-21st century (2030-2059) under the RCP8.5 scenario; and (d) late-21st century (2070-2099) under the RCP8.5 scenario. Results are shown in a grid size of approximately 25 km × 25 km. A value of 1 indicates no changes in the designed precipitation, a value of 1.2, for example, means a factor of 1.2 is suggested to apply to the designed precipitation intensity.

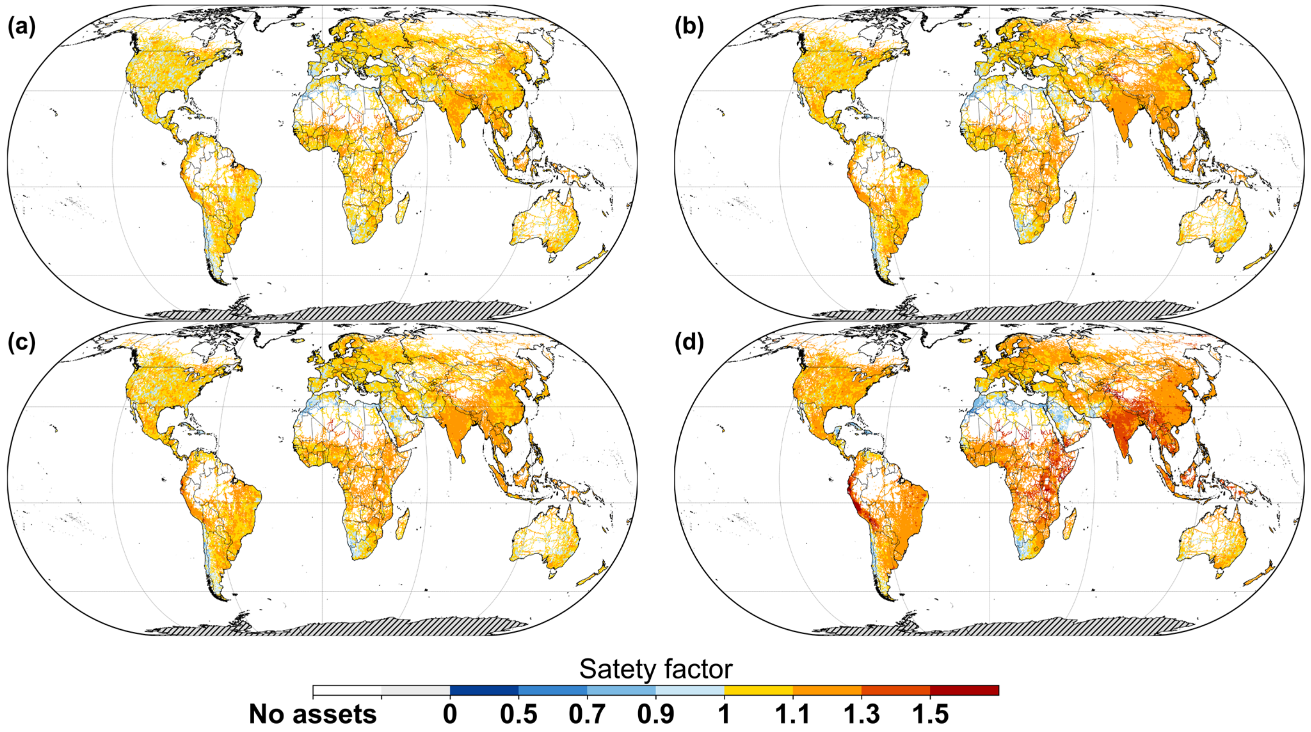

Supplementary Fig.22. Safety factor for climate change adaptation with respect to current design under different time periods and scenarios (based higher current design standards assumption): (a) mid-21st century (2030-2059) under the RCP4.5 scenario; (b) late-21st century (2070-2099) under the RCP4.5 scenario; (c) mid-21st century (2030-2059) under the RCP8.5 scenario; and (d) late-21st century (2070-2099) under the RCP8.5 scenario. Results are shown in a grid size of approximately 25 km × 25 km. A value of 1 indicates no changes in the designed precipitation, a value of 1.2, for example, means a factor of 1.2 is suggested to apply to the designed precipitation intensity.
