# Supplementary material for: Global transportation infrastructure exposure to the change of precipitation in a warmer world
Source: Nat Commun. 2023 May 3;14:2541. doi: 10.1038/s41467-023-38203-3 (PMC10156714; doi:10.1038/s41467-023-38203-3)
Supplement: Supplementary file 3 — Description of additional supplementary files [file 41467_2023_38203_MOESM3_ESM.pdf]

<u>Description of additional supplementary files</u>

Supplementary Data 1 : exposure inventory aggregated by countries.
